# Understanding how we age: insights into inflammaging

**DOI:** 10.1186/2046-2395-2-8

**Published:** 2013-05-02

**Authors:** Daniel Baylis, David B Bartlett, Harnish P Patel, Helen C Roberts

**Affiliations:** 1 Department of Medicine for Older People, University Hospital Southampton, Southampton, UK; 2 Academic Geriatric Medicine, University of Southampton, Southampton, UK; 3 MRC-ARUK Centre for Musculoskeletal Ageing Research, University of Birmingham, Birmingham, UK

**Keywords:** Ageing, Cortisol, DHEAS, Frailty, Inflammaging, Inflammation, Older people

## Abstract

Inflammaging is characterized by the upregulation of the inflammatory response that occurs with advancing age; its roots are strongly embedded in evolutionary theory.

Inflammaging is believed to be a consequence of a remodelling of the innate and acquired immune system, resulting in chronic inflammatory cytokine production.

Complex interrelated genetic, environmental and age-related factors determine an individual’s vulnerability or resilience to inflammaging. These factors include polymorphisms to the promoter regions of cytokines, cytokine receptors and antagonists, age-related decreases in autophagy and increased adiposity. Anti-inflammaging describes the upregulation of the hypothalamic-pituitary axis in response to inflammaging, leading to higher levels of cortisol, which in turn may be detrimental, contributing to less successful ageing and frailty. This may be countered by the adrenal steroid dehydroepiandrosterone, which itself declines with age, leaving certain individuals more vulnerable. Inflammaging and anti-inflammaging have both been linked with a number of age-related outcomes, including chronic morbidity, functional decline and mortality. This important area of research offers unique insights into the ageing process and the potential for screening and targeted interventions.

## Review

### Introduction

The immune system protects an organism from disease. The first reference to immunity dates back to the plague of Athens in 430 BC when it was noticed that people who recovered from the disease could then nurse others without contracting it a second time. It is recognized that the immune system, comprising both innate (nonspecific) and acquired (specific) components, is an intricate defence system that is highly conserved across vertebrate species, and has, from an evolutionary perspective, undergone strong pressures to maximize survival to allow procreation. The significant improvements in human survival and lifespan to well beyond childbearing ages have been totally ‘unpredicted’ by evolution. As a consequence, human immune systems are exposed to considerable additional antigenic exposure outside the forces of natural selection. It is in this situation that immunity begins to exert negative effects on human ageing (antagonistic pleiotropy), leading to gradual systemic failures [1,2].

The immune system of older people declines in reliability and efficiency with age, resulting in greater susceptibility to pathology as a consequence of inflammation, for example, cardiovascular disease, Alzheimer's disease, autoreactivity and vaccine failure, as well as an increased vulnerability to infectious disease [3-5]. These changes are further compounded by reduced responsiveness and impaired communication between all cells of the immune system. The overall change to the immune system with age is termed immunosenescence and has a multifactorial aetiology; a consequence of the complexity of the immune system as well as of multiple genetic and environmental influences [3]. Immunosenescence of the innate immune system is primarily characterized by reduced cellular superoxide production and capability for phagocytosis. Involution of the thymus and reduced responsiveness to new antigen load, owing to reduced naïve: memory cell ratio and expansion of mature cell clones, characterizes immunosenescence of the acquired immune system (Table 1).

**Table 1:** **Age-related changes to the immune system, adapted from **[3]

**Immune system**	**Cell type**	**Age–related changes**
Innate immunity	Neutrophils	Reduced phagocytic ability of opsonized bacteria and impaired superoxide production
	Monocytes or macrophages	Reduced levels of MHC class II complexes, reduced phagocytic ability and impaired superoxide production
	Dendritic cells	Impaired capability to phagocytose apoptotic cells; impaired migration
	Natural killer cells	Reduced cytotoxicity
Acquired immunity	T cells	Thymus atrophy
		Reduced naïve cells leaving thymus, severely contracted T-cell repertoire after 70 years
		Impaired expansion and differentiation
		Increased proinflammatory cytokine release, reduced IL-2 production
		Increased memory and effector cells
		Impaired T-cell help of B cells
		Reduced regulatory T cells; possible increased inflammation and autoreactivity
		Expanded clones of herpes virus (for example, cytomegalovirus) CD8+ cells, dominating the T-cell repertoire and limiting response to other pathogens
	B cells	Reduced number of mature B cells leaving bone marrow
		Increased memory B cells, decline in naïve B- cells
		Reduced responsiveness to stimulatory molecules
		Impaired antibody response to vaccination

*MHC*, major histocompatibility complex.

Research into age-related changes of the immune system is gathering pace as its importance within the context of multiple pathologies in ageing populations is realized. As part of this advance, Franceschi and colleagues [6] described the phenomenon of ‘inflammaging’ at the turn of the millennium as part of the spectrum of immunosenescence. Inflammaging denotes an upregulation of the inflammatory response that occurs with age, resulting in a low-grade chronic systemic proinflammatory state. It is characterized by raised levels of proinflammatory cytokines interleukin-1 (IL-1), interleukin-6 (IL-6) and tumour necrosis factor (TNF); all of which have been shown to rise with age [7] and be involved in the pathogenesis of most age-associated diseases [8]. C-reactive protein (CRP), an acute phase protein produced by the liver in response to IL-6, is also a useful marker of inflammaging, more commonly used in clinical practice, as well as a robust predictor of risk for cardiovascular and other diseases [9,10].

### Inflammaging

**Figure 1:**
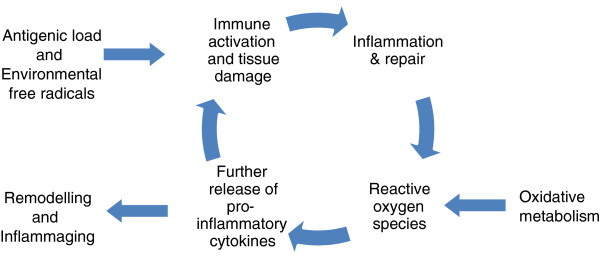
Cycle of inflammaging.

Inflammaging is believed to be a consequence of a cumulative lifetime exposure to antigenic load caused by both clinical and subclinical infections as well as exposure to noninfective antigens [1]. The consequent inflammatory response, tissue damage and production of reactive oxygen species that cause oxidative damage also elicits the release of additional cytokines, principally from cells of the innate immune system [11] but also from the acquired immune response. This results in a vicious cycle, driving immune system remodelling and favouring a chronic proinflammatory state where pathophysiological changes, tissue injury and healing proceed simultaneously. Irreversible cellular and molecular damage that is not clinically evident slowly accumulates over decades (Figure 1).

Immunosenescence of the acquired system has received a great deal of attention in recent years and is by a cellular ‘exhaustion’, which can contribute to inflammaging. Thymic output is reduced with age resulting in reduced T-cell repertoire and increased oligoclonal expansion of memory and effector-memory cells [12]. This imbalance results in a reduced ability to clear novel pathogens (prolonging infection duration) as well as an increase in functionally distinct T-cell populations, which have an amplified proinflammatory phenotype [13]. The CD8^+^ T cell population is altered to a greater extent than the CD4^+^ population and is associated with specificity to single antigens, particularly latent viral infections, such as cytomegalovirus, Epstein-Barr and varicella zoster virus. Increases in numbers of antigen-specific cells with age are associated with an increase in the number of terminally differentiated ‘senescent’ cells, which occupy a large proportion of immune space. These cells, particularly CD8^+^, are extremely potent producers of inflammatory cytokines and have been heavily associated with reduced antiviral immunity and inflammatory related pathologies [14]. With age, these antigen-specific cells do not proliferate as well, and on mitogenic and viral stimulation produce more IL-6 and TNF than their younger counterparts [15]. Subsequently antigenic load is a known driver of immunosenescence and has been associated with increased IL-6 and mortality [16]. Although it was recently shown that cytomegalovirus infection did not drive inflammaging (IL-6, TNF and CRP) in healthy older people over a 10-year period, the study could not account for other latent infections, suggesting a combined role of other antigenic stimulus in systemic inflammaging [17]. Therefore, T-cell immunosenescence is characterized by an increased proinflammatory phenotype and most probably contributes to inflammaging in a manner dependent on previous exposure to and reactivation of antigenic challenges and exhaustion of T-cell repertoire.

Of the innate immune system, monocytes and macrophages are suggested to contribute to inflammaging more than any other cell type. Monocyte changes with age contribute to inflammaging by a functional shift towards a proinflammatory phenotype and reduced function [18]. Monocytes consist of three distinct subtypes, differentiated by expression of CD14 and CD16; CD14^++^/CD16^−^ (classical), CD14^++^/CD16^+^ (intermediate) and CD14^+^/CD16^++^ (nonclassical), with varying degrees of functional capabilities [19]. The CD16^+^ positive monocytes constitutively produce more IL-6, IL-1β and TNF basally and with stimulation, with older people having a significantly larger proportion of CD16^+^ cells than younger people [20,21]. Furthermore, the CD16^+^ population have increased adherence and migrate towards endothelial lesions via CX3CR1, contributing to increased atheroma plaque formation [22]. Subsequently, monocytes can contribute to inflammaging by chronically increased production of inflammatory cytokines and prolongation of the immune response.

**Figure 2:**
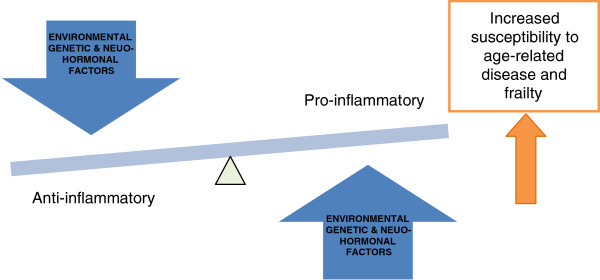
Relationship between pro- and anti-inflammation and ageing.

A two-hit hypothesis states that the extent to which an individual is vulnerable to inflammaging and its physiological consequences is dependent first on the degree of increased proinflammatory environment and then on the resilience of that individual to withstand it. This is reflected in part by the balance of increasing levels of proinflammatory cytokines with anti-inflammatory cytokines, and also concentrations of soluble cytokine receptors and cytokine receptor antagonists. These are believed to affect the degree to which an individual is susceptible to an antigenic load, the rate at which age-related pathologies progress and individual vulnerability to frailty [2,7] (Figure 2). Indeed, centenarians, a population deemed to have aged successfully, have strong and effective anti-inflammatory responses with a reduced proinflammatory capacity [23]. This degree of susceptibility is multifactorial and a consequence of complex genetic and environmental interactions as well as of the ageing process itself.

### Factors influencing susceptibility to inflammaging

There is now convincing evidence that susceptibility to inflammaging is, in part, genetically determined through polymorphisms of promoter regions of genes that either affect cytokine production rates or alter their protein structure to produce a functional variant. For example, polymorphisms in the promoter C/G 174 on the IL-6 gene have been shown to affect serum IL-6 concentrations [24]. Similar associations have been found in alleles for IL-10 [25] and polymorphisms of the toll-like receptor 4 residing on the membrane of dendritic cells. Macrophages positively affect inflammatory responses and are associated with an increased risk of acute myocardial infarction [26].

Telomeres may also be implicated; these are protein complexes made from several thousand repetitive DNA sequences located at the end of chromosomes to protect them from deterioration or fusion. Telomere repetitive sequences shorten with each cell division, eventually reaching a critical number leading to cellular senescence and death. There is evidence that this process is associated with inflammation, although this is controversial, and the direction of this relationship is unclear and could be bi-directional. For example, raised IL-6 and TNF was associated with shorter telomere length in 1,962 individuals of the Health, Ageing and Body Composition Study [27]. Shorter telomere length has also been found in cohorts of patients with chronic inflammatory diseases of liver, kidney and lung [28]. Negative relationships with telomere length have also been demonstrated with CRP [29] and IL-6 [30].

Ageing is associated with decreases in autophagy mechanisms, further contributing to proinflammatory environments [31]. Autophagy is a cellular housekeeping mechanism that is responsible for the removal of dysfunctional intracellular protein (for example, dead organelles, damaged scaffold proteins) via lysomal degradation. This prevents the activation of inflammasomes; intracellular multi-protein sensors that stimulate the inflammatory response after recognizing danger signals emanating from proteins, such as the intracellular danger-associated molecular patterns that occur as a consequence of either tissue injury or necrosis. The consequence of a decline of autophagy with age is therefore an increased activation of the inflammasome and greater proinflammatory responses [32].

Ageing, though often visually characterized by a decrease in subcutaneous tissue and sarcopenia [33], is associated with a linear accumulation of adipose tissue, both around the viscera and via the fatty infiltration of several organs, including liver, bone and muscle. This adipose tissue, once thought of as inert, is now considered a major endocrine and paracrine organ [34]; to date, approximately one hundred adipokines have been identified, falling into several functional groups [35]; many are proinflammatory. For example, the classic adipokine, leptin, not only has a primary role in energy homeostasis but also causes the stimulation and differentiation of monocytes into macrophages, activates NK-lymphocytes and induces the production of a number of proinflammatory cytokines, including TNF and IL-6 [36]. Consequently, greater degrees of adiposity are directly asso ciated with greater proinflammatory environments, partly via proinflammatory adipokines and also from immune cells residing within adipose tissue.

### Anti-inflammaging

As with all complex organisms, single biological systems rarely work in isolation. There is extensive cross-talk between the immune and endocrine axes, which, together with the neural system, form the major communication network in the body [37].

Cortisol is a glucocorticoid hormone secreted by the adrenal gland in response to pituitary secretion of adrenal corticotrophic hormone, which itself is stimulated by corticotrophin releasing hormone from the hypothalamus; together these form the hypothalamic-pituitary-adrenal (HPA) axis. Cortisol plays key roles in the stress response and is also immunosuppressive [38]. Neuronal cells within the HPA axis contain multiple cytokine receptors, particularly for IL-1, IL-6 and TNF [39], and it has been demonstrated in human beings *in vivo* that injection of IL-6 or TNF induces a marked change in HPA axis [40]. Therefore, an inevitable physiological response to inflammaging is an increase in circulating cortisol levels [41].

This mechanism has been termed *anti-inflammaging* and although it represents an appropriate attempt to counter the inflammaging process it may also have negative implications. These include the paradox of both inflammaging and the global immunosuppression seen with increasing age, as well as associations with frailty via catabolic effects on several tissue types, such as liver (gluconeogenesis), muscle (protein catabolism) and bone (resorption) [41].

Dehydroepiandrosterone (DHEA) and its sulphated precursor, DHEA sulphate (DHEAS), have opposing actions to cortisol and may protect individuals from the negative effects of anti-inflammaging. They are secreted from the adrenal cortex and, in smaller amounts from the testis and ovary, although subsequently converted to sex steroids in the latter; they account for 30% to 50% of testosterone in men and 100% of oestrogen in post-menopausal women. Like cortisol, DHEAS is also secreted in response to adrenal corticotrophic hormone but serum levels are more stable as a consequence of stronger binding to albumin [42]. Circulating DHEAS concentrations reach a maximum in early adulthood and subsequently decline with age to approximately 10% to 20% of maximal levels by 70 years [43]. There is large interindividual variability in circulating DHEAS concentrations; some post-menopausal women have barely detectable serum concentrations whilst others have normal values. The fall in DHEAS is accompanied by a parallel fall in androgens, resulting in women with low DHEAS levels experiencing a deficit of sex steroids for their remaining lifetime and negative implications for the ageing process [43].

More recently, an immunomodulating role for DHEAS has been realized [38]; low levels are associated with chronic inflammatory conditions and the molecule counteracts the effects of cortisol via antagonism of glucocorticoid receptors, either directly or via its downstream metabolites. Further, DHEAS may directly inhibit glucocorticoid receptor production. This importance as a counterbalance to cortisol has been demonstrated in a number of *in-vitro* studies where the immunosuppression of neutrophil function that occurs with cortisol has been successfully overcome by co-incubation with DHEAS [44]. Additionally, a number of studies have consistently demonstrated the steroid to be an enhancer of IL-2 secretion from T-helper 1 (Th1) cells whilst negatively affecting secretion from T-helper 2 (Th2) cells. Therefore, it has been suggested that observed declines in DHEAS levels with age, at least in part, explains the cytokine dysregulation seen with age and specifically the shift towards Th2 cytokine profiles.

**Figure 3:**
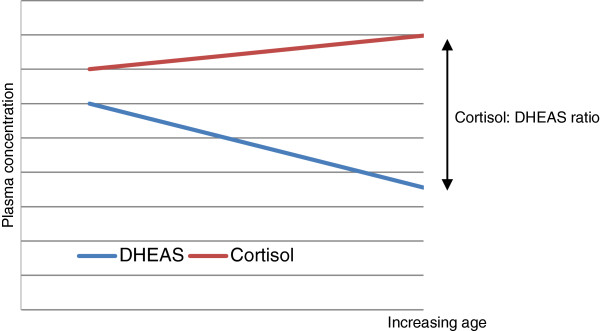
Representation of changes in adrenocorticoid hormones with age.

From this discussion it is apparent that both cortisol and DHEAS have opposing effects relating to the immune system. Cortisol causes immune-suppression and its concentration increases with age, whilst DHEA(S) antagonizes the effects of cortisol and is immune modulating, and its concentration falls with age. Therefore, when considering the effects of the HPA axis on inflammaging, the ratio of cortisol to DHEAS (Figure 3) may more accurately reflect the HPA axis than either cortisol or DHEAS alone. An *in-vivo* study of cortisol and DHEAS in patients with septic shock after a hip fracture showed that higher cortisol levels, lower DHEAS levels, and a higher cortisol: DHEAS ratio correlated with poorer clinical outcomes [45]. This ratio has also been positively associated with metabolic syndrome and all-cause, cancer and other medical cause mortality in Vietnam veterans [38,46].

Cortisol: DHEAS ratios have the potential for manipulation via DHEAS supplementation. To date, DHEAS supplementation has been trialled in a number of chronic inflammatory conditions, usually with nonbeneficial outcomes. However, doses used have always been low. The only true beneficial effect of DHEAS supplementation has been seen in patients with systemic lupus erythematosus, where the drug successfully reduces disease activity and the number of exacerbations [47-49].

### Associations with chronic disease and ageing

Inflammaging, in particular elevations in levels of TNF, IL-6, IL-1 and CRP, are strong independent risk factors for morbidity and mortality in older people [50]. Epidemiological and mechanistic studies suggest that many age-related diseases are initiated or worsened by systemic inflammation, including neurodegeneration and atherosclerosis [51,52]. For example, proinflammatory cytokines have been shown to interact with the processing and production of Aβ peptide, the pathological hallmark feature of Alzheimer’s disease, and autopsy studies have shown that the brains of patients who had Alzheimer’s disease have dramatically higher levels of proinflammatory markers than high-pathology controls who have pathological features of Alzheimer’s disease without its symptoms [53].

Inflammaging also affects the anabolic-catabolic balance within myocytes, causing a shift towards catabolism, atrophy and progression of sarcopenia; this is a major contributor to functional decline and frailty. As with other age-related disease processes, it is believed that the degree of shift towards catabolism affects the rate of sarcopenic decline, and cachexia in older people may represent this process *in extremis*. Proinflammatory cytokines appear to play a particularly important role through activation of NF-κB and the ubiquitin system which is implicated in myofibre proteolysis [54]. It may be that interindividual variations in vulnerability to inflammaging in part explain the different rates at which individuals become sarcopenic. A number of community-based observational studies have demonstrated associations between proinflammatory cytokine profiles and sarcopenia. For example, cross-sectional analysis from the InCHIANTI study (1,020 men and women over 65 years) demonstrated a significant association between inflammation (IL-6, IL-1R, CRP) and both poor physical performance and reduced muscle strength [55]. Longitudinal associations also exist. Schaap used data from 2,177 men and women, (mean age 73), to show significant associations between TNF and 5-year change in muscle mass [56]. Associations with sarcopenia have also been demonstrated in the Framingham Heart Study (IL-6) [57] and the Longitudinal Aging Study Amsterdam (IL-6, CRP) [58] and have recently undergone systematic review [59]. Inflammaging is also implicated in the progression of osteoporosis [60] and dementia [51]; both strongly implicated in the frailty syndrome.

Higher cortisol levels have been associated with increased mortality in patients with stroke [61], sepsis [62], heart failure [63] and sarcopenia [64]. Low levels of DHEA have been demonstrated in patients with chronic inflammatory diseases, including inflammatory bowel disease, rheumatoid arthritis, systemic lupus erythematosus and pemphigus [65]. Moreover, associations have also been established between low DHEAS concentrations and cardiovascular disease, sarcopenia, osteoporosis and all-cause mortality [66-68].

The cumulative consequence of having a greater degree of inflammaging and anti-inflammaging is increased susceptibility to, and a faster progression of, all age-related diseases. This results in an increased vulnerability to stressors and reduced functional ability and is associated with the development of the frailty syndrome [50]. Interestingly, a 10-year study of 254 healthy, community-dwelling older people demonstrated that those individuals with baseline markers of greater inflammaging (white cell count) and anti-inflammaging (cortisol to DHEAS ratio) were significantly more likely to be frail at 10-year follow-up. Intriguingly, the authors speculate that this might offer a tool to screen for people who are ‘less robust’ in late middle age to allow early intervention before functional decline and the development of frailty [50].

## Conclusions

Inflammaging is a pathological phenomenon and a central concept that brings together our understanding of age-related chronic disease, functional decline and frailty across the lifecourse. It is a consequence of lifelong exposure of the immune system to antigenic stimuli and complex genetic, environmental and age-related mechanisms that expose varying degrees of vulnerability or resilience. An important consequence of inflammaging is activation of the HPA axis, anti-inflammaging and a widening of the gap between molar concentrations of cortisol and DHEA(S). This ‘double-edged’ sword provides an appropriate counter to inflammaging but also has multi-system consequences in terms of disease progression and immunosuppression.

As populations age, a better understanding of these processes is essential to identify older people who are at risk of ageing-related chronic diseases, in order to facilitate successful, early, targeted interventions.

## Abbreviations

CRP C: Reactive protein; DHEAS: DHEA sulphate; HPA: Hypothalamic-pituitary-adrenal; IL: Interleukin; MHC: Major histocompatibility complex; NIHR: National Institute of Health Research; Th: T-helper; TNF: Tumour necrosis factor

## Competing interests

The authors’ declared that they have no competing interest.

## Authors’ contributions

DB and HPP prepared the first draft and DBB added immunological input. All authors read and approved the final draft.

## Authors’ information

DB is supported by a National Institute of Health Research (NIHR) Doctoral Research Fellowship. DBB is supported by a Biotechnology and Biological Sciences Research Council grant. HPP is supported by the University of Southampton National Institute of Health Clinical Lectureship Scheme. HCR is a Senior Clinical Lecturer in Geriatric Medicine at the University of Southampton.
